# Frequency, symptoms and outcome of intestinal metastases of bronchopulmonary cancer. Case report and review of the literature

**DOI:** 10.1186/1477-7800-2-13

**Published:** 2005-06-06

**Authors:** Andreas Hillenbrand, Joern Sträter, Doris Henne-Bruns

**Affiliations:** 1 Department of Visceral and Transplantation Surgery, University of Ulm, Steinhövelstr. 9; 89075 Ulm; Germany; 2 Department of Pathology, Klinik Esslingen, 73730 Esslingen a. N.; Germany

**Keywords:** Pulmonary Cancer, small bowel metastases

## Abstract

**Background:**

We report a new case of small bowel metastases from primary lung cancer. Such metastases are not exceptional, but their clinical manifestations are rare.

**Case presentation:**

The case involved a 56-year-old man with a squamous cell carcinoma of the lung (stage IV) that had been treated with chemotherapy. He presented fourteen months after diagnosis with an acute abdominal pain. Abdominal CT-scan demonstrated a perforated jejunum and he underwent emergency surgery. Postoperative pathologic analysis confirmed the diagnosis of metastatic pulmonary carcinoma. The patient was discharged after ten days, but died 8 weeks after surgery at home on tumor progression.

**Conclusion:**

We were able to find 58 documented similar cases in the literature. Most cases presented with bowel perforation or obstruction. Squamous cell carcinoma was the most common histological cell type followed by large cell carcinoma. Other metastases are often present, and the prognosis is mostly fatal at short term.

## Background

Lung cancer is the most common cause of cancer related death in both men and women in Western countries. More than 25% of all cancer deaths are attributable to lung cancer [1,2]. Intraabdominal metastases are not exceptional, but their clinical manifestations are rare [3]. We report such a case presenting with small bowel perforation.

## Case Report

A 56-year-old man (varnisher by profession, heavy smoker with 160 pack years) presented in September 2002 with a five month history of cough, fever, night-sweat and a weight loss of 5 kg in 12 months. A bronchoscopy with biopsy revealed a squamous cell carcinoma of the lung. A FDG-PET/CT scanning showed a disseminated praetracheal, aortoal, supraclavicular and infracarinal lymphadenopathy and a spleen metastasis (stage IV) [4]. The patient received six cycles of Cisplatinum-Gemcitabine chemotherapy, followed by a second line Mitomycine-Vinorelbine polychemotherapy. A re-evaluation after one year revealed liver metastases and increasing mediastinal lymphadenopathy, followed by additionally Taxotere polychemotherapy. A brain magnetic resonance imaging after a pathologically Romberg Test in December 2003 showed a left hemisphere brain metastasis.

At New Year's Day 2004, the patient presented again with a three days history of increasing abdominal pain, diffuse abdominal guarding and rebound tenderness. Laboratory evaluation was without pathological findings except a marked leukocytosis (WBC = 24,000/mm^3^).

**Figure 1:**
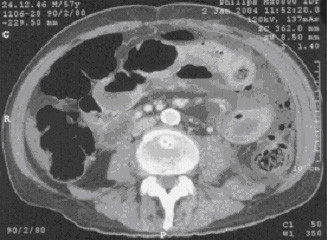
CT scan with intravenouse contrast: Free intraperitoneal air and a enlarged jejunum.

**Figure 2:**
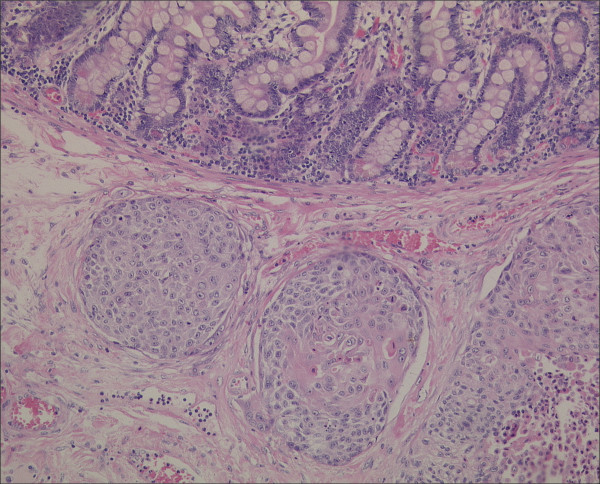
Small bowel metastasis; Stain: Hematoxylin & Eosin (amplification 1:62).

Abdominal CT-scan displayed free intraperitoneal air and a perforated jejunum (Figure 1). The patient was operated on with the diagnosis of perforated jejunum. At surgery, a 2 cm mass was found at the surface of a loop of the jejunum with perforation of 1 cm in diameter, furthermore left upper quadrant peritonitis with pus, stool and a fibrin coating. The affected segment of the jejunum was resected with an end-to-end anastomosis and an abdominal lavage was performed. Postoperative pathologic analysis confirmed the diagnosis of metastatic pulmonary carcinoma (Figure 2). The surgical margins were negative for tumor. Postoperatively, the patient was given a regular diet on postoperative day four and discharged home fourteen days after surgery. The patient died 8 weeks after surgery at home on tumor progression.

## Conclusion and review of the literature

Despite the early and widespread dissemination of lung carcinoma to many body sites, clinically significant metastases in the small bowel are rare and typically occur only in the advanced stage of the disease [5]. But metastases to the gastrointestinal tract from lung cancer may not be as rare as thought according to autopsy series.

Antler et al. report in an autopsy series of 423 cases of primary tumors of the lung gastrointestinal tract involvement in 58 cases (14%) [6]. Out of these 58 cases, 14 were caused by direct extension of the tumor from the lung. 44 cases (10%) had distant metastasis without contact of the primary tumor to the gastrointestinal tract. The most common site of metastasis was the oesophagus (29 cases), followed by ileum (13 cases) and stomach (10 cases).

Mc Neill et al. reported in their autopsy study of 431 cases small/large bowel metastases without oesophageal/stomach metastases in 46 cases (11%) [7]. All patients with small bowel metastasis had at least one other site of metastatic disease with an average of 4.8 sites.

In an autopsy study of Burbige et al. 18 out of 147 (12%) patients with a diagnosis of primary carcinoma of the lung had metastatic lesions in the gastrointestinal tract [8]. Twelve of the 18 patients (67%) had signs or symptoms suggesting gastrointestinal involvement but only four were diagnosed premortem [8].

Mc Neill et al. and Antler et al. [6,7] report in their autopsy studies, the most common histologic type of lung tumor causing gastrointestinal tract metastasis was squamous cell, followed by large cell carcinoma as shown in Table 1. In all three autopsy studies large cell carcinoma is relative to the tumor incidence prone to small bowel metastasis [6-8].

**Table 1:** Incidence of cell type causing gastrointestinal (Antler et al.) or small/large bowel (McNeill et al.) metastases

Histologic cell type	Antler et al. 1982 [6]: 58 gastrointesinal metastasis out of 423 cases	McNeill et al. 1987 [7]: 46 small/large bowel metastasis out of 431 cases
Squamous cell	19 out of 173 (11%)	15/5 out of 199 (10%)
Adenocarcinoma	6 out of 72 (8%)	13/6 out of 108 (18%)
Large cell	17 out of 87 (20%)	12/6 out of 31 (58%)
Small Cell	11 out of 48 (23%)	6/5 out of 73 (15%)
Others	5 out of 43 (11%)	0/2 out of 20 (10%)

Reviewing the literature between 1961 and 2003 we were able to document 58 clinically manifest metastasis to the small bowel [3,7,9-37]. One report even describes eleven cases in a thirteen year period in a single institution. This result strongly suggests that metastasis to the small bowel may not be as rare as thought.

Over 80% of patients with metastases to the small bowel were male, with ages ranging from 36 to 78 years with a median of 60 years.

Metastases to the small bowel may present as perforation (34/58; 59%), obstruction (17/58; 29%), six (10%) patients had haemorrhage, and in one patient (2%) the small bowel metastasis was discovered in a staging CT scan.

Of those patients with known location of metastasis (n = 38), 21 (55%) had jejunal metastasis, 12 (32%) ileal, three (8%) duodenal and two (5%) patient had both jejunal and ileal metastasis.

In 34 patients we were able to document the histological tumor type. Squamous cell carcinoma was the most common histological cell type (17/34; 50%) followed by large cell carcinoma (8/34; 24%) and adenocarcinoma (7/34; 21%).

In 7 (12%) patients the initial diagnose of lung cancer was discovered because of the abdominal symptoms, before any pulmonary disease was considered. Five out of these seven patients presented with abdominal perforation. This fact could retract the statement that particularly chemotherapy induces necrosis of the tumor cells which has replaced a portion of the small bowel wall, thus causing perforation of abdominal metastasis [21,23]. According to McNeill et al. lung cancer metastasis have a greater tendency to undergo necrosis compared with other malignant tumor metastasis [7]. Therefore, there may be a greater tendency for these metastases to cause perforation before attaining enough bulk to cause obstruction [7].

Phillips et al. [38] reviewed the literature for indications for laparotomy in 27 patients presenting with isolated small bowel metastases from extra-abdominal primary sites in a 22 year time period. The author found only six small bowel perforations and all six were in patients with carcinomas of the lung as primary site.

Small bowel metastasis typically occurs in the final states of widespread disease [7].

Surgery in this group of patients will have a high morbidity and mortality due to cancer, age and associated medical problems [36]. Of those patients who were known to have died (n = 39), mean postoperative survival was 3 weeks. Nine (9/39; 23%) patients died within the first week, 25 (25/39; 64%) within the first month, four patients however were alive more than one year after surgery.

Review of the literature indicates that metastases of primary pulmonary carcinoma to small bowel occur late in the course of the disease, are more common than thought and may be associated with serious clinical complications. When symptomatic, patients usually present with a small bowel perforation, obstruction or haemorrhage. Squamous cell carcinoma and large cell carcinoma of the lung are more likely to result in gastrointestinal metastases than the other cell types.

Treatment of obstructed or perforated small bowel is similar to any acute surgical abdomen: prompt exploratory laparotomy. When perforation of small bowel lesions is found, the procedure of choice is resection of the involved small intestine with primary enteroenterostomy, obstructions could be treated with bypass procedures.

## List of abbreviations

FDG-PET/CT:Fluorodeoxyglucose-Positron Emission Tomography/Computed Tomography

CT-scan: Computed Tomography

WBC: White blood cell count

## Competing interests

No reimbursements, fees, funding or salary from an organization were received. Furthermore I have no other financial or non-finacial competing interests.
